# Opsismodysplasia: Phosphate Wasting Osteodystrophy Responds to Bisphosphonate Therapy

**DOI:** 10.3389/fped.2015.00048

**Published:** 2015-06-22

**Authors:** Ansab Khwaja, Shawn E. Parnell, Kathryn Ness, Viviana Bompadre, Klane K. White

**Affiliations:** ^1^Orthopedics and Sports Medicine, Seattle Children’s Hospital, University of Washington, Seattle, WA, USA; ^2^Department of Radiology, Seattle Children’s Hospital, University of Washington, Seattle, WA, USA; ^3^Division of Endocrinology, Department of Pediatrics, Seattle Children’s Hospital, University of Washington, Seattle, WA, USA

**Keywords:** opsismodysplasia, metabolic bone disease, skeletal dysplasias, scoliosis, genu varum, bisphosphonates

## Abstract

We present two siblings affected with opsismodysplasia (OPS), a rare skeletal dysplasia caused by mutations in the inositol polyphosphate phosphatase-like 1 gene. The skeletal findings include short stature with postnatal onset micromelia, marked platyspondyly, squared metacarpals, delayed skeletal ossification, metaphyseal cupping, and postnatal micromelia. Respiratory compromise, delayed ambulation, and progressive lower extremity deformities are described. The severity of findings is variable. Renal phosphate wasting is associated with severe bone demineralization and a more severe phenotype. This report represents the first described cases of opsismodysplasia treated with intravenous bisphosphonate (pamidronate). Surgical management for lower extremity deformities associated with OPS is also reviewed.

Level of Evidence: IV Case series

## Introduction

Opsismodysplasia (OPS) is a rare autosomal recessive skeletal dysplasia associated with delayed bone maturation and micromelia (1, 2). Maroteaux et al. originally coined the term in 1984 (3). The most frequent clinical signs evident at birth are relative macrocephaly with a large anterior fontanelle, craniofacial abnormalities including a prominent brow, depressed nasal bridge, a small anteverted nose, and a relatively long philtrum, abdominal protrusion, and hypotonia (4–6). The main radiological features are severe platyspondyly, short long bones including squared metacarpals, delayed epiphyseal ossification, and metaphyseal flaring and cupping (7–10). Square iliac wings with horizontal acetabular roof and ossification defect at the skull base are also seen (11).

Opsismodysplasia can be associated with renal phosphate wasting (12). Mutations in the inositol polyphosphate phosphatase-like 1 (*INPPL1*) gene were recently identified to be a cause OPS (5, 9, 13). The two patients in this case report were diagnosed with opsismodysplasia based on their clinical and radiographic findings. Their diagnosis was confirmed on molecular analysis. The older sibling had findings of renal phosphate wasting. Both were treated with a bisphosphonate (pamidronate). This case series adds to the current literature on this rare condition with a clinical description of our two patients, documenting their surgical management as well as their response to bisphosphonate therapy. Our study is IRB approved (12259) and the patient’s family gave full consent.

## Case 1

**Figure 1:**
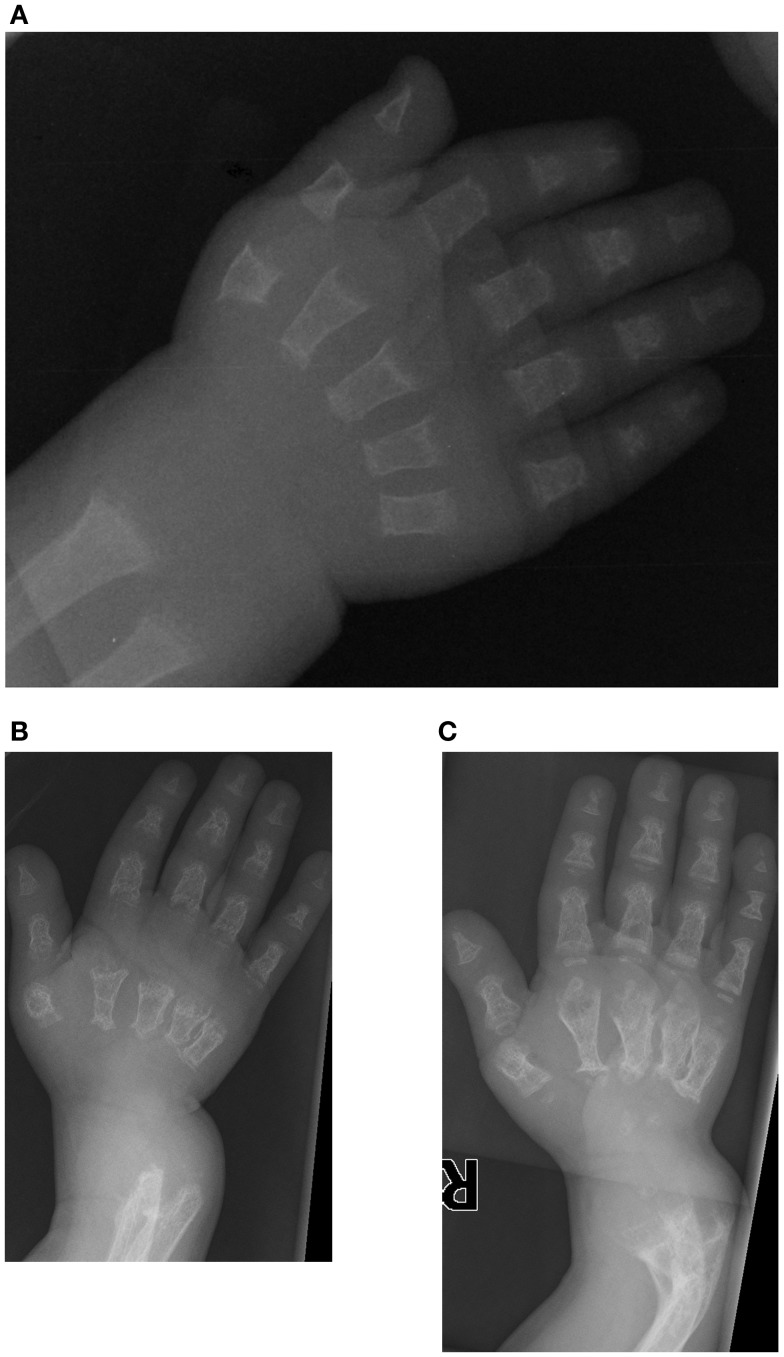
**Case 1, AP view of right hand demonstrates delayed carpal ossification, shortened phalanges and metacarpals, and metaphyseal cupping at (A) 14 months of age, (B) at 3 years 5 months of age with decreased bone mineralization and (C) at 4 years 4 months of age following pamidronate therapy**. Note the bowing of the distal radius and ulna.

The first patient is a male who was initially evaluated at 14 months of age for a skeletal dysplasia. He was referred to the genetics clinic because of his short stature, and presented with a relatively large orbitofrontal cortex and fontanelle but no other chronic medical illnesses. Radiographs from a skeletal survey obtained at 14 months of age revealed short tubular bones with delayed epiphyseal ossification and metaphyseal cupping (Figure 1), shallow acetabulae with bilateral hip subluxation, and short femoral necks with metaphyseal irregularity. There was also frontal bossing in the skull and diffuse osteopenia. At follow up at 16 months of age his height and weight were at the fifth percentile (64.2 cm and 7.9 kg). He had a large and soft anterior fontanelle for his age, a depressed nasal bridge and prominent forehead. He was unable to fully extend his elbows and had bilateral anterolateral tibial bowing. His abdomen was soft without organomegaly.

At 2 years of age his head circumference was in the 75th percentile (49.8 cm), with height at 5th percentile (69.1 cm) and weight at 5th percentile (8.72 kg), reinforcing the previously described phenotype of relative macrocephaly without hydrocephalus. His examination revealed volar subluxation of the carpus, and a sharp bow of the distal radius. He lacked 30° of elbow extension. There was similar bowing at the distal end of tibia with the foot subluxated posteriorly. He had limited hip abduction to 40° bilaterally and full knee extension. He did not have significant kyphosis or scoliosis, but a somewhat barrel-shaped chest. Calcium and magnesium levels were normal, but phosphate was low (2.0 mg/dl).

**Figure 2:**
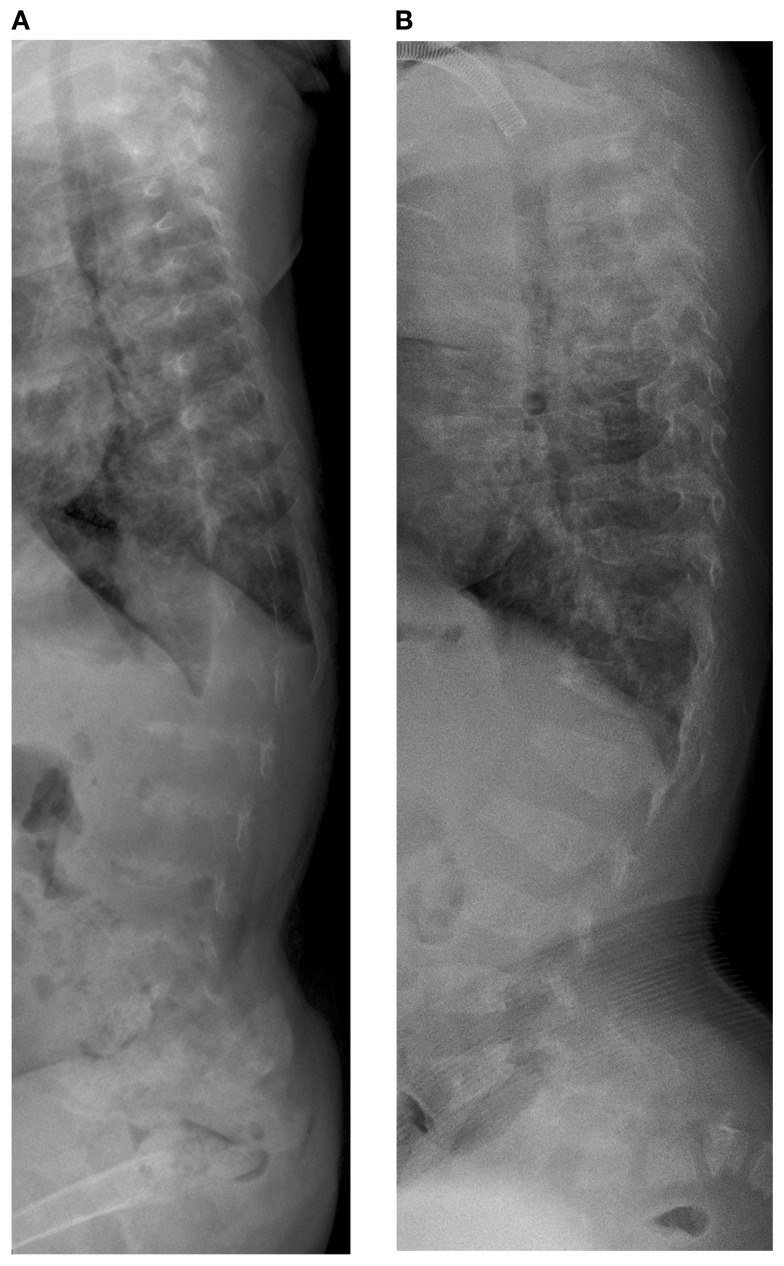
**Case 1, (A) Lateral spine shows marked platyspondyly at 3 years 5 months, (B) which improves on follow up films at 7 years 6 months of age**.

**Figure 3:**
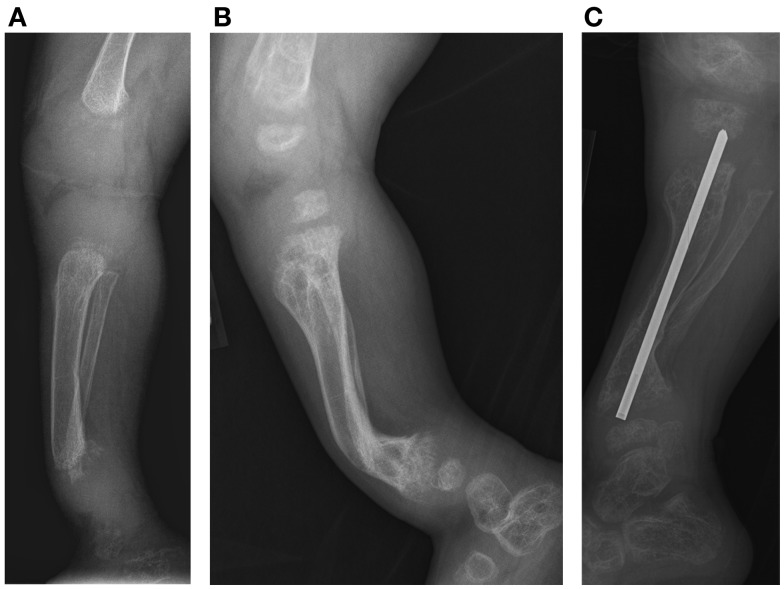
**Case 1, (A) Lateral view of tibia demonstrates initial poor mineralization and irregularity of the distal tibial metaphysis with anterior bowing at 2 years 4 months of age, (B) with improved mineralization but persistent bowing at age 4 years 4 months and (C) subsequent osteotomy and intramedullary pin placement at 8 years 7 months**.

Initial skeletal survey revealed diffuse cervical, thoracic, and lumbar platyspondyly without significant scoliosis or kyphosis. Repeat skeletal survey at 2 years and 10 months of age (9/19/05) and again at 3 years and 5 months (4/10/06) demonstrated marked platyspondyly (Figure 2) and also demonstrated more severe, now marked osteopenia and new tapered appearance of the proximal metacarpals, distal radius and ulna, and femoral necks. Periosteal reaction was present along the shafts of the left humerus, radius, and ulna, without discrete fracture lines. There was continued significant delay in epiphyseal ossification, with none present at the hips, shoulders, elbows, and carpal rows. Dedicated radiographs of the left tibia and fibula demonstrated anterior convex bowing of the distal leg with associated tapered appearance of metadiaphyses, and no significant distal epiphyseal ossification (Figure 3). There was also very poor midfoot and hindfoot ossification (3/14/05 tib fib).

At approximately 2½ years of age, the patient developed progressive respiratory failure secondary to his skeletal dysplasia and was started on supplemental oxygen. He was identified to have cardiomegaly with enlargement of the left ventricle. He was found to have a hypoplastic right kidney. His bone age was severely delayed and estimated at between 0 and 3 months of age. Dual x-ray absorptiometry (DXA) scan at this point showed severe demineralization of bones, and was he was started on pamidronate for his osteoporosis. He received 0.5 mg/kg/IV infusion on each of 3 days, given sequentially. At 3 years of age, he underwent a tracheostomy and required mechanical ventilatory support for progressive respiratory failure. A gastrostomy-tube was placed for nutritional support.

After the first pamidronate infusions (two sets of 3 day infusions), whole-body bone mineral density was increased by 11.6% and total lumbar spine bone mineral density showed a 26.9% increase. He continued to receive pamidronate infusions quarterly at 9 mg/kg/year and undergoing DXA scans annually. (Table 1) At 4 years of age, with the noted clinical improvement and reversal in his deteriorating pulmonary function associated with his pamidronate infusions, he started limited ambulation. He was provided with physical therapy services. At 5½ years old, he was able to take six steps independently and 20–30 steps with a walker. At 6 years, he was able to walk 15–20 feet independently. He underwent bilateral Sofield osteotomies of the tibiae to straighten his legs at 6 years of age. With this procedure, he had a 3-month pause in his pamidronate infusions. At 8½ years, he was walking 100 feet at school with a walker. At 9 years his pamidronate infusion was reduced to 1 mg/kg/infusion twice yearly. At 10 years of age, he has stopped taking his wheelchair to school and runs during recess, demonstrating a remarkable progress presumably related to his pamidronate usage.

**Table 1:** **Patient 1 bone mineral density**.

Age (years)	Whole body (g/cm^2^)	Z-score	Lumbar (g/cm^2^)	Z-score	Left hip bone (g/cm^2^)	Z-score	Right Femur (R1) (g/cm^2^)	Z-score
4	0.505	−3.1	0.162	−6.3	0.459			
6	0.527	−3.2	0.147	−6.7	0.392	−3.4	0.352	−3.4
7^a^	0.619	−1.8	0.198	−5.9			0.350	−3.4
8	0.609	−2.6	0.201	−5.8	0.382	−4.1	0.327	−3.3
9	0.580	−3.6	0.317	−4.0	0.490	−2.9	0.382	−3.1
10			0.659	+0.5	0.804	+0.5	0.482	−2.5

*^a^S/P bilateral Sofield osteotomies*.

His diagnosis of opsismodysplasia was recently confirmed by exome sequencing analysis. He was identified to have homozygosity for a missense mutation in exon 17 of the INPPL-1 gene (c.1976C > T).

## Case 2

**Figure 4:**
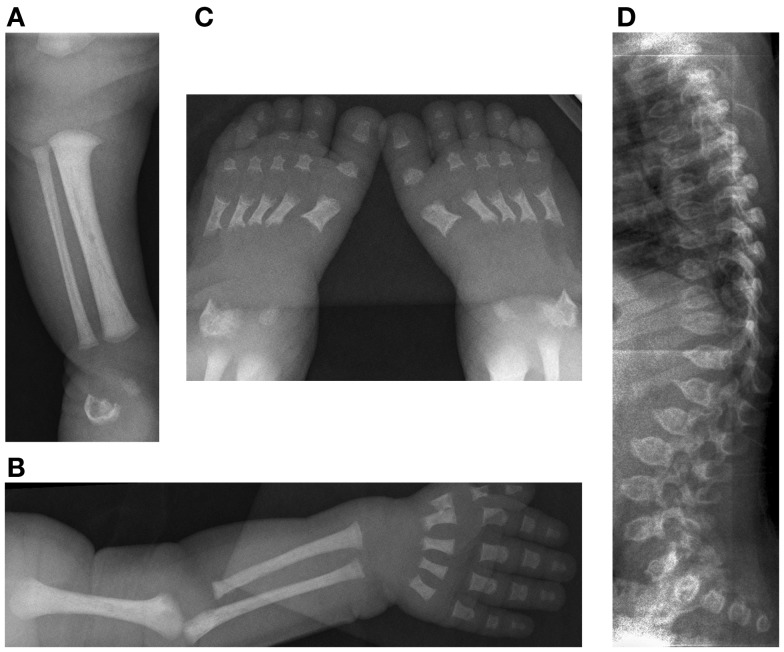
**Case 2, Initial skeletal survey at 9 days of life (A) [10/09/08] demonstrates lack of visualization of the distal femoral and proximal tibial ossification centers, (B) relatively short long bones of the left upper extremity and (C) feet with metaphyseal cupping and irregular ossification of the talus and calcaneus**. The degree of platyspondyly **(D)** is less than that of her sibling on lateral spine radiographs.

The younger sibling of the patient in Case 1 also had clinical, radiological, and molecular findings consistent with a diagnosis of opsismodysplasia. She was born at 38 weeks via C-section and at 9 days of life presented with respiratory distress and a right-sided pneumothorax. She was discovered to have a large atrial septal defect (ASD). Because of her brother’s skeletal dysplasia, she was evaluated for the diagnosis of opsismodysplasia. Radiographically, no secondary ossification centers were identified, specifically to include the proximal tibial and distal femoral epiphyses, which should be present at this age. (Figure 4) The metacarpals and phalanges of her hands and feet were shortened with metaphyseal cupping. Platyspondyly was present and the symphysis pubis was poorly ossified. Her talus and calcaneus were ossified, but irregular in shape. Her ribs were mildly shortened with cupped anterior rib ends. Her serum phosphorous was normal.

At the age of 9 days, on physical examination she measured 48 cm (25th–50th percentile), weighed 3.06 kg (25th percentile), and had a head circumference of (of 36.5 cm (75th percentile). Her anterior fontanelle was flat and soft and measured 5.5 × 6.0 (greater than 90th percentile). At 9 months her length was 62 cm and her weight 6.3 kg. These are both below the third percentile for her age. She was also diagnosed with restrictive lung disease, and she had a gastrostomy-tube placed because of feeding difficulties. Her head circumference at 13 months however, was 90th percentile while her height and weight remained at below the third percentile.

**Figure 5:**
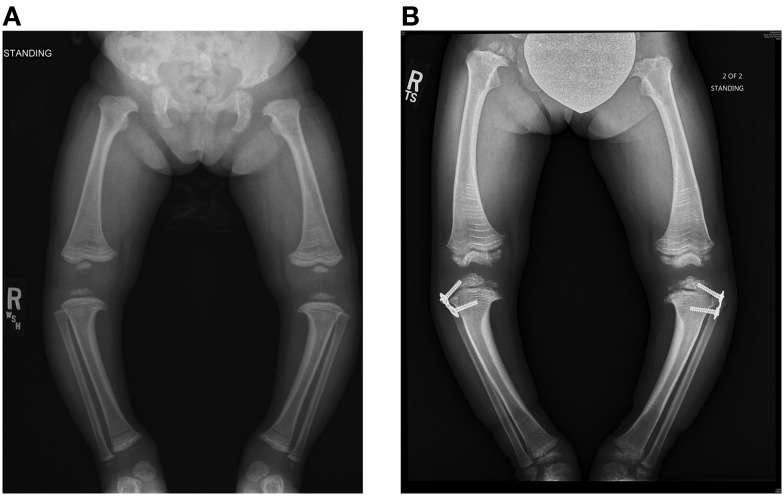
**Standing frontal projection of bilateral lower extremities (A) at age 3 years and 2 months (12/05/11) demonstrates bilateral genu varum, which was treated with bilateral lateral hemiepiphysiodesis plate and screws (B) of the proximal tibia, as shown on the right in**.

A DXA scan was performed at 1 year and revealed diminished bone mineral density of the left femur (0.334–0.326 g/cm^2^) from her 5-month scan (no *Z* scores available because of age – Table 2). Because of her decreased mineral density, she was started on pamidronate at a dose of 0.5 mg/kg/infusion for a total of 9 mg/kg/year pamidronate. She was standing and walking at 2 years of age. She had slight anterolateral bowing of her distal tibias. She was identified to have scoliosis with a 42° right thoracic curve, and a 57° left lumbar curve. She developed a mild bilateral genu varum deformity by age three and still had lack of ossification of the proximal femoral. (Figure 5) Cervical spine films show odontoid hypoplasia, with no evidence of atlantoaxial or occipitocervical instability. She had continued diffuse platyspondyly with anterior pointing of the vertebral bodies.

**Table 2:** **Patient 2 bone mineral density**.

Age (years)	Whole body (g/cm^2^)	*Z*-score	Lumbar (g/cm^2^)	*Z*-score	Left hip bone (g/cm^2^)	*Z*-score	Right femur (R1) (g/cm^2^)	*Z*-score
1/2^a^			0.168				0.289	
1^a^			0.213				0.223	
2			0.474				0.485	
3			0.605	+2.9	0.671		0.672	1.3
4	0.702	+2.4	0.665	+3.0	0.683		0.643	+0.3

*^a^Limited lumbar spine BMD*.

A follow-up DXA scan at approximately 3 years of age revealed her lumbar spine bone mineral density to be normal. Her pamidronate infusions were reduced to once every 6 months for a dose of 2 mg/kg/year. At 4 years, she had significant genu varum and underwent placement of lateral proximal tibial growth modulation plates. She continues to have normal to increased bone mineral density compared with the reference population. Exome sequencing for this child also revealed homozygosity for a missense mutation in exon 17 of the INPPL1 gene (c.1976C > T).

## Discussion

We present two siblings with OPS who presented with variability in their clinical and radiographic findings. Both were identified to have homozygosity for a missense mutation in exon 17 of the INPPL1 gene (c.1976C > T). The clinical presentation and radiographic findings in Patient 1 were much more severe. He had phosphate wasting, progressive bony demineralization, and respiratory failure. Both patients’ clinical presentations are largely in accordance with the literature. Their facial features were consistent with those described in OPS, including a prominent brow, relative macrocephaly with a large anterior fontanel, depressed nasal bridge, anteverted nose, and a long philtrum (4–6). Both had short limbs, lower extremity deformity, and small hands and feet. They also developed respiratory complications, as has also been reported in the literature (6). Patient 1 was more severely affected, which we hypothesize was related to his phosphate wasting. His lack of treatment until a later age is another possible contributory factor.

On radiographs, our patients corroborate the literature, which reports delayed epiphyseal ossification, platyspondyly, metaphyseal cupping, and short metacarpals and phalanges (7–10). Both patients had anterolateral bowing of the tibias, patient 1 requiring surgical intervention. These cases provide evidence of the variability in severity of findings in siblings with opsismodysplasia and suggest that those with phosphate wasting have more severe skeletal findings and respiratory compromise. Both of our patients also had difficulty feeding, which is not reported in the literature to date. This is the first known report of pamidronate therapy for opsismodysplasia, and the surgical management of the lower extremity deformities associated with this disorder.

As with other disorders, such as polyostotic fibrous dysplasia, we found with these two patients that phosphate wasting is associated with a poorer outcome. Both patients demonstrated clinical progress and improvement in bone mineral density measured using serial DXA scan studies with pamidronate therapy. Surgical correction by osteotomy appears to have lasting results. It is too soon to determine whether growth modulation techniques will be of benefit in patient 2.

Surgical management of these patients mirrored treatment well described for other metabolic bone disorders such as osteogenesis imperfecta and rickets. When enough growth is available, guided growth techniques may be appropriate, although the ultimate outcome of patient 2 is still pending. For the more complex deformities seen in patient 1, single level, or in this case, multi-level Sofield realignment osteotomies may be required. Our patient ultimately began to grow off the William’s rod that was used, so the use of newer generation telescoping rods may now present a better option in these circumstances. Another interesting technical note is that due to the poor ossification of the metaphyses and epiphyses, location of the rod ends can be challenging with fluoroscopy.

## Summary

In summary, we describe two siblings diagnosed with opsismodysplasia based on clinical and radiographic findings, confirmed by genetic analysis. The older sibling had a more severe clinical course and renal phosphate wasting. His significant associated lower extremity deformities were corrected with Sofield osteotomies. After 7 years of pamidronate therapy, he no longer requires daytime ventillatory support and his ambulatory function has improved such that he requires no longer requires assistive devices.

The younger sibling’s showed no signs of urine phosphate wasting. Due to her diagnosis and low bone mineral density, she was initiated on pamidronate at the age of 1 year. Her bone mineral density has normalized when compared to the reference population. She has genu varum that is currently being treated with growth modulation plates.

## Conflict of Interest Statement

The authors declare that the research was conducted in the absence of any commercial or financial relationships that could be construed as a potential conflict of interest.

## References

[1] Online Mendelian Inheritance in Man, OMIM^®^. Baltimore, MD: Johns Hopkins University (2013). Available from: http://omim.org/

[2] Tyler K Sarioglu N Kunze J. Five familial cases of opsismodysplasia substantiate the hypothesis of autosomal recessive inheritance. Am J Med Genet (1999) 83(1):47–52. 10076884 10.1002/(sici)1096-8628(19990305)83:1<47::aid-ajmg9>3.0.co;2-5

[3] Maroteaux P Stanescu V Stanescu R Le Marec B Moraine C Lejarraga H. Opsismodysplasia: a new type of chondrodysplasia with predominant involvement of the bones of the hand and the vertebrae. Am J Med Genet (1984) 19(1):171–82. 10.1002/ajmg.1320190117 6496568

[4] Al Kaissi A Chehida FB Ghachem MB Grill F Klaushofer K. Atlanto-axial segmentation defects and Os odontoideum in two male siblings with opsismodysplasia. Skeletal Radiol (2009) 38(3):293–6. 10.1007/s00256-008-0623-4 19050869

[5] Below JE Earl DL Shively KM McMillin MJ Smith JD Turner EH Whole-genome analysis reveals that mutations in inositol polyphosphate phosphatase-like 1 cause opsismodysplasia. Am J Hum Genet (2013) 92(1):137–43. 10.1016/j.ajhg.2012.11.011 23273567 PMC3542462

[6] Santos HG Saraiva JM Opsismodysplasia: another case and literature review. Clin Dysmorphol (1995) 4(3):222–6. 10.1097/00019605-199507000-00005 7551158

[7] Chai EC Singaraja RR Opsismodysplasia: implications of mutations in the developmental gene Inppl1. Clin Genet (2013) 83(6):527–9. 10.1111/cge.12136 23464704

[8] Cormier-Daire V Delezoide AL Philip N Marcorelles P Casas K Hillion Y Clinical, radiological, and chondro-osseous findings in opsismodysplasia: survey of a series of 12 unreported cases. J Med Genet (2003) 40(3):195–200. 10.1136/jmg.40.3.195 12624139 PMC1735387

[9] Huber C Faqeih EA Bartholdi D Bole-Feysot C Borochowitz Z Cavalcanti DP Exome sequencing identifies Inppl1 mutations as a cause of opsismodysplasia. Am J Hum Genet (2013) 92(1):144–9. 10.1016/j.ajhg.2012.11.015 23273569 PMC3542463

[10] Ramos FJ González JP Cortabarria C Domenech E Pérez-González J Bueno M. A further case of opsismodysplasia with hydrocephalus. Eur J Med Genet (2006) 49(1):93–100. 10.1016/j.ejmg.2005.04.002 16473316

[11] Lachman RS Taybi and Lachman’s Radiology of Syndromes, Metabolic Disorders and Skeletal Dysplasias. 5th ed Philadelphia: Mosby (2007). 1013 p.

[12] Zeger MD Adkins D Fordham LA White KE Schoenau E Rauch F Hypophosphatemic rickets in opsismodysplasia. J Pediatr Endocrinol Metab (2007) 20(1):79–86. 10.1515/JPEM.2007.20.1.79 17315533

[13] Iida A Okamoto N Miyake N Nishimura G Minami S Sugimoto T Exome sequencing identifies a novel Inppl1 mutation in opsismodysplasia. J Hum Genet (2013) 58(6):391–4. 10.1038/jhg.2013.25 23552673

